# The Functional Alterations in Top-Down Attention Streams of Parkinson’s disease Measured by EEG

**DOI:** 10.1038/s41598-018-29036-y

**Published:** 2018-07-13

**Authors:** Hye Bin Yoo, Edgar Omar de la Concha, Dirk De Ridder, Barbara A. Pickut, Sven Vanneste

**Affiliations:** 1 Lab for Clinical & Integrative Neuroscience, School of Behavioral and Brain Sciences, The University of Texas at Dallas, Richardson, TX USA; 2 Department of Surgical Sciences, Section of Neurosurgery, Dunedin School of Medicine, University of Otago, Dunedin, New Zealand; 3 Department of Neurology, Mercy Health St. Mary’s Hospital, Grand Rapids, MI USA

## Abstract

Early and moderate Parkinson’s disease patients seem to have attention dysfunctions manifested differentially in separate attention streams: top-down and bottom-up. With a focus on the neurophysiological underpinnings of such differences, this study evaluated source-localized regional activity and functional connectivity of regions in the top-down and bottom-up streams as well as any discordance between the two streams. Resting state electroencephalography was used for 36 Parkinson’s disease patients and 36 healthy controls matched for age and gender. Parkinson’s disease patients showed disproportionally higher bilateral gamma activity in the bottom-up stream and higher left alpha2 connectivity in the top-down stream when compared to age-matched controls. An additional cross-frequency coupling analysis showed that Parkinson’s patients have higher alpha2-gamma coupling in the right posterior parietal cortex, which is part of the top-down stream. Higher coupling in this region was also associated with lower severity of motor symptoms in Parkinson’s disease. This study provides evidence that in Parkinson’s disease, the activity in gamma frequency band and connectivity in alpha2 frequency band is discordant between top-down and bottom-up attention streams.

## Introduction

Parkinson’s disease (PD) is a chronic neurodegenerative disorder characterized by symptoms of motor dysfunction such as rigidity, tremors, postural imbalance, slowness in movement (bradykinesia), and dysfunctions in voluntary movement (akinesia)^1,2^. PD also involves cognitive impairment that appears in the early, often premotor, phase of the disease^3,4^ and significantly diminishes the quality of life for approximately 50–60% of PD patients^5^. Cognitive impairments associated with PD are mainly related to executive functions, such as working memory and attention^6,7^. Attentional dysfunction, in particular, is an important predictor of both a decreased quality of life^8^ and the later onset of dementia^9^ in PD.

Attention can be divided into two separate streams: a dorsal, top-down network and a ventral, bottom-up network^10^. The top-down network is associated with the voluntary, goal-directed allocation of attention to certain features, objects, or regions in space. The bottom-up network is primarily stimulus-driven and is activated when salient stimuli attract attention, and is, therefore, considered a circuit breaker^11^. Top-down regions include the posterior parietal cortices and the frontal eye fields, while bottom-up regions include the temporoparietal junctions and the ventrolateral prefrontal cortices^11^. These networks are identified in both task-based^11^ and resting state neuroimaging paradigms^12^ and are related to attentional functions^13^, even in resting state conditions^14^. Top-down control appears to be selectively impaired in early and moderate stages of PD, whereas bottom-up attentional control processes remain mostly intact^15,16^, indicating a functional discordance between the two streams. Furthermore, a recent study led to the hypothesis that PD patients have problems with internal attentional control, which leads to the excessive guidance of behavior by external cues, laying dependency on these cues to a larger extent than healthy subjects^17^. However, the neurophysiological mechanism underlying these differences in attentional controls is not yet clear.

In electroencephalography (EEG), alpha (8–12 Hz) and gamma (>30 Hz) frequency bands seem to play distinct roles in attention^18–20^. Alpha band oscillations selectively deactivate task-irrelevant functions to re-allocate attention towards goal-directed behavior^20–22^ and inhibit the intrusion of bottom-up information^23,24^. In the resting state, alpha band maintains the alert state by better directing one to an attention-demanding cue^25,26^. Specifically, the lower alpha frequency range (alpha1: 8–10 Hz) appears to be associated with the broad-focused demand of attention when handling the input of information, while the higher alpha frequency range (alpha2: 10–12 Hz) is narrow-focused and more important for a specific demand in processing task-related information^21,27–29^. A recent review suggests that broad- and narrow-focused attention in top-down control stream are distinguished from each other and affect different psychiatric disorders^30^, exemplifying the need to analyze alpha1 and alpha2 bands separately. On the other hand, an increase in gamma band oscillations is likely to represent enhanced attention^31,32^. Gamma band activity appears to be dependent on the orientation of attention to external stimuli^33–35^. Alpha and gamma seem to interact upon attentional demand to provide selective top-down attention^36–38^. Modulation of alpha oscillatory activity is also accompanied by an increase in gamma band activity, which has been associated with the deployment of attention^33,34,39^. From these previous studies, we hypothesize that alterations in alpha and gamma frequency bands underlie the discordance between top-down and bottom-up attention streams in PD.

This study aimed to understand the neurophysiological underpinnings of top-down attention in early to moderate medicated PD using source-localized EEG, focusing on alpha and gamma frequency bands. As an overview, we performed a whole-brain activity analysis for alpha1, alpha2, and gamma bands to identify changes without applying a priori hypotheses. In subsequent analyses, we utilized regions of interest in the frontoparietal resting state network implicated in top-down and bottom-up attention streams^11^. Coordinates were selected based on previous literature that investigated the recruitment of top-down and bottom-up attention during a surprise stimulus task^40^. Although these regions were based on functional magnetic resonance imaging, previous studies noted that such resting state networks can also be identified using neurophysiological signals^41,42^, citing the spatial similarity of attention streams in EEG^43^. The discordance between top-down and bottom-up attention streams were investigated in terms of the regional activity level and the functional connectivity weights. It is hypothesized that in PD, alpha connectivity within top-down attention stream would increase, resulting in an excess of inhibitory modulation^44^. Consequently, gamma activity level would likely decrease in the top-down stream, indicating that PD patients have less activated top-down attention than healthy controls^17^.

Based on our analyses of functional discordance, this study further investigated the cross-frequency coupling between alpha and gamma to discern the implications of changes in the functional connectivity. Cross-frequency coupling quantifies the degree of effective signal transmission^45^. It may represent the active modulation of attention across frequency bands, and higher coupling would likely be beneficial for PD patients.

## Methods

**Figure 1:**
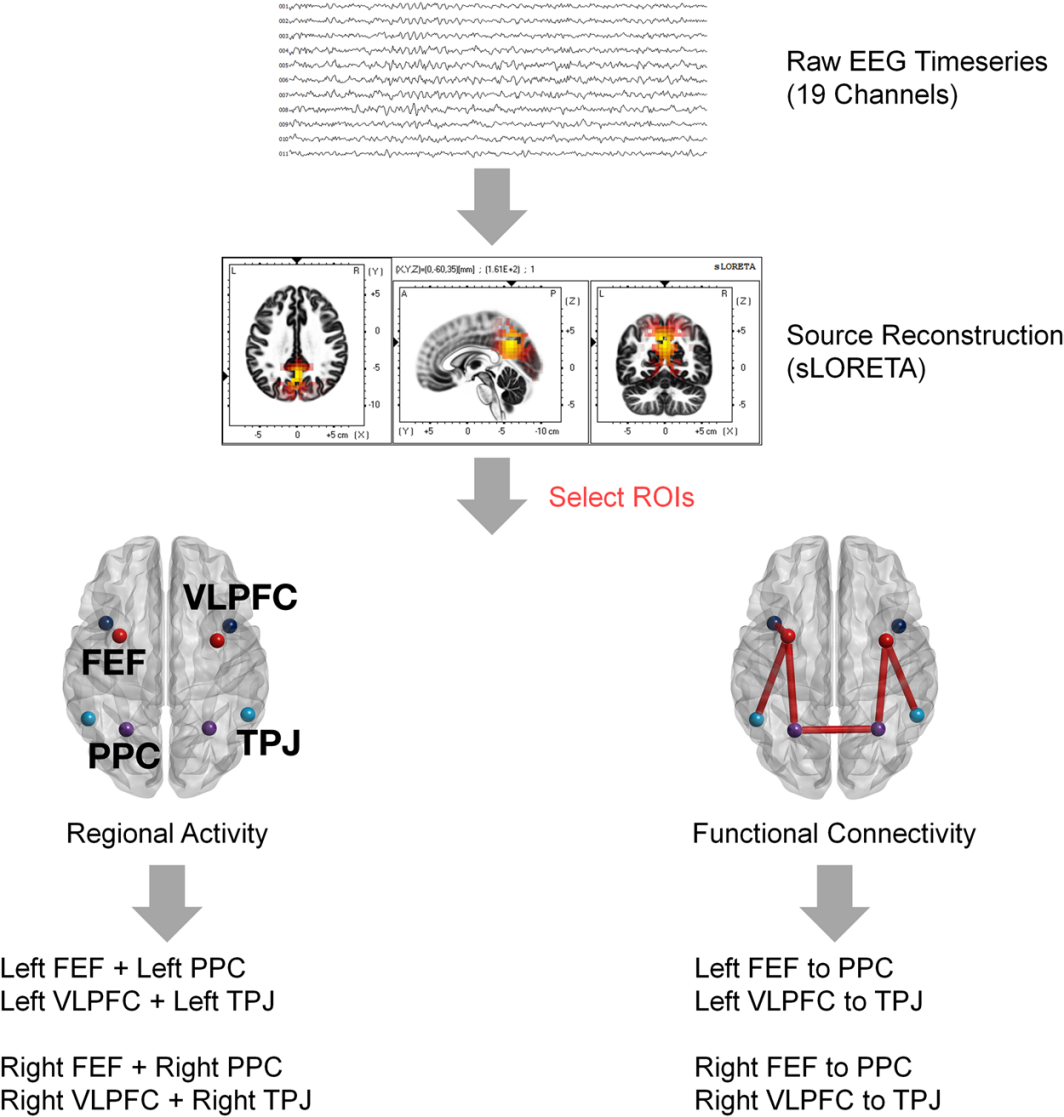
Graphical demonstration of the methods applied in this study. The voxel-based whole-brain analysis firstly confirmed the increased activity near the attention networks. Secondly, regions of interest analysis was performed for regional activity and functional connectivity. For the further analyses, regional activity values were summed together for each attention stream, and hemisphere. Functional connectivity measured a bidirectional coherence between the time series of two regions involved in each attention stream of one side.

The graphical representation of data processing is shown in Fig. 1.

### Study participants

The local ethical committee of the University Hospital Antwerp, Belgium, approved the study protocol. All research was performed in accordance with the approved guidelines and regulations. Experimenters informed all participants about the purpose and the procedure of the study and received signed consent from subjects, in accordance with the Declaration of Helsinki (2000). The control group consisted of 36 participants without any neurological or psychiatric conditions, who were also free of neurological and psychiatric pharmaceuticals. The same number of PD outpatients were recruited from the Neurology Clinic of the University Hospital Antwerp, Belgium. The PD participants were all on a stable dose of all medications, which was unchanged for at least 30 days prior to the EEG, in order to avoid any tremors during the recordings that could induce motion-related artifacts or interfere with the theta band recording^46^ and potentially compromise the imaging analyses. We included participants in accordance with the following criteria: (1) diagnosed as PD according to the UK Brain Bank Criteria; (2) diagnosed with Hoehn and Yahr (H-Y) stages 1–3; (3) lack of features suggestive of atypical parkinsonism; (4) no records of using drugs that may induce parkinsonism 60 days prior to inclusion; (5) lack of cognitive dysfunction measured by Montreal Cognitive Assessment Test (that is score ≥26); (6) lack of known unstable or life-threatening concomitant diseases.

### Behavioral measures

The participants were assessed by a neurologist specialized in movement disorders on the Unified PD Rating Scale (UPDRS)^47^, while they were in a stably medicated state. There were four subscales in the UPDRS counted separately: (1) Mentation, behavior and mood (1–4); (2) Activities of daily living (5–17); (3) Motor examination (18–31); (4 A) Complications of therapy – dyskinesia (32–35); (4B) Complications – clinical fluctuations (36–39); (4 C) Complications – others (40–42). Among these, the scores in the UPDRS subscales 1–3 were analyzed for correlation to neurophysiological measures. The demographics and the behavioral information of all subjects is shown in Table 1.

**Table 1:** Demographic information.

	CON (Mean ± SD)	PD
Age	62.00 ± 9.03	62.17 ± 9.09
Gender (F %)	50% (18 in 36)	50% (18 in 36)
PD assessments		
H-Y	n/a	2.29 ± 0.38
UPDRS 1 (1–4) Mentation, Behavior and Mood	n/a	2.33 ± 2.53
UPDRS 2 (5–17) Activities of Daily Living	n/a	10.22 ± 5.39
UPDRS 3 (18–31) Motor Examination	n/a	27.42 ± 10.22
UPDRS 4 A (32–35) Complication – Dyskinesia	n/a	1.28 ± 1.73
UPDRS 4B (36–39) Complication – Fluctuations	n/a	1.44 ± 1.63
UPDRS 4 C (40–42) Complication – Others	n/a	0.75 ± 0.94
UPDRS Total	n/a	43.44 ± 15.53

Abbreviations: H-Y = Hoehn-Yahr scale, UPDRS = Unified Parkinson’s Disease Rating Scale.

### EEG recording

Participants were instructed not to drink alcohol 24 hours prior to the EEG recording to avoid alcohol-induced changes in the EEG results^48^, and caffeinated beverages on the day of the recording to avoid caffeine-induced alpha and beta power decreases^49,50^. The vigilance of participants was checked by monitoring EEG streams on the screen and watching for patterns such as slowing of alpha rhythm or the appearance of spindles, in order to prevent possible enhancement of the theta power due to drowsiness^51^. No participants included in the current study showed such drowsiness-related EEG changes.

The resting state EEGs were recorded for at least 5 minutes at 19 scalp sites of a Tin-electrode cap (ElectroCap, Ohio, United States), using a Mitsar amplifier (EEG-201) and the WinEEG software version 2.84.44 (Mitsar, St. Petersberg, Russia, http://www.mitsar-medical.com). The functional image was acquired in a lighted room, shielded from sound and stray electric fields, with each participant sitting upright with eyes closed. The raw EEG was collected for 100 2-second epochs, and the sampling rate was 1024 Hz. The data was acquired using 19 electrodes in the standard 10/20 International placement referenced to linked ears, and impedances were maintained below 5 kΩ on all electrodes throughout the EEG recording. Data was band-pass filtered at the higher boundary of 200 Hz and the lower boundary of 0.15 Hz. The data was then resampled to 128 Hz and a further band-pass filter (fast Fourier transform filter applying Hanning window) within the 2–44 Hz window was applied. Data was then imported into the Eureka! Software^52^, plotted, and carefully inspected manually for any artifacts. All episodic artifacts including eye blinks, eye movements, teeth clenching, and muscle movements were manually removed.

### Source reconstruction

Standardized low-resolution brain electromagnetic tomography (sLORETA), a functional imaging method that yields standardized current density (A/m^2^), based on certain electrophysiological and neuroanatomical constraints, was utilized for the analysis of EEG signals. It estimated the intracerebral sources generating the scalp-recorded electrical activity in each of the three decomposed frequency bands of interest: alpha1 (8–10 Hz), alpha2 (10–12 Hz), and gamma (30.5–44 Hz). In the statistical analysis, sLORETA corrected for multiple comparisons regarding the number of electrodes, voxels, time samples, and the discrete frequency bands^53,54^.

SLORETA estimates the exact three-dimensional source of the band-pass filtered signals that come from the electrodes spread over an EEG cap. The positions of the electrodes are calculated in Cartesian coordinates on the MNI brain, which are derived from the normalization of the international 10/5 system^55^. Other than this information, sLORETA utilizes two major clues for solving the ill-posed problem: standard positions of the fiducial points of the head using a realistic head model, with spatial restriction onto cortical gray matter and hippocampi as defined by digitized MNI152 template^56^, and minimum-norm least squares constraints for the current distribution on the brain^57^. Further, sLORETA applies weight to the signal depending on the depth of the estimated source and the constraints it is given^53^. It accounts for two sources of variance, which are from actual sources and noisy measurements and are independent of each other, and distinguishes the actual one. Its solution space consists of 6,239 voxels (voxel size: 5 ∗ 5 ∗ 5 mm^3^) for the whole brain.

### Regions of interest

Regions were selected based on the frontoparietal resting state network, which is well-implicated in attention functions^11^. This study, utilized the bilateral posterior parietal cortex and the frontal eye field for top-down regions, and the temporoparietal junction and the ventrolateral prefrontal cortex for bottom-up regions, chosen based on previous literature that investigated the recruitment of top-down and bottom-up attention streams during a surprise stimulus task^40^. MNI coordinates for the regions of interest (ROIs) were defined based on areas corresponding to the regions identified in a surprise stimulus task that activated both bottom-up and top-down attention streams in a previous study^40^. Regarding the spatial resolution of reconstructed EEG signals, all ROIs were placed in distances that surpass the maximum margin of error, which is 2 ∗ 5 ∗  √3 = 17.3025 mm.

In the source solution space of the whole brain, the activity was evaluated by taking the decimal logarithm-transformed electric current density per voxel (log(A/m^2^)). ROI-based activity level was calculated by averaging the current density across all voxels that belong to predefined ROIs (Table 2). For the further analyses, activity levels of ROIs in each attention stream were added to represent the activity of the stream.

**Table 2:** Regions of interest in connectivity analysis.

Region	Side	MNI (x, y, z)	Functions
Posterior Parietal Cortex	Left	−26.2, −59.05, 49.89	Top-down
Posterior Parietal Cortex	Right	28.84, −58.21, 44.38	Top-down
Frontal Eye Field	Left	−30.69, 3.14, 49.41	Top-down
Frontal Eye Field	Right	34.11, −0.03, 46.38	Top-down
Temporoparietal Junction	Left	−51.41, −51.96, 23.84	Bottom-up
Temporoparietal Junction	Right	54.5, −49.15, 25.12	Bottom-up
Ventrolateral Prefrontal Cortex	Left	−39.67, 11.56, 25.2	Bottom-up
Ventrolateral Prefrontal Cortex	Right	42.45, 9.72, 23.98	Bottom-up

Abbreviations: MNI = Montreal Neurological Institute template.

### Functional connectivity

Functional connectivity was measured as the linear dependence between time series of ROIs, using the connectivity toolbox of sLORETA across all frequency bands. In order to minimize the physiological influence of volume conduction^58^, only the lagged linear coherence between ROIs was quantified, excluding zero-lag contributions^59,60^. The connectivity values were zero only when the signals were independent of each other.

### Statistical analysis of whole-brain activity

For the analysis of source-reconstructed EEG signals, statistical non-parametric mapping with a permutations test was used to differentiate the current density between the groups (independent *t*-test)^54^. The statistical test counted for 5,000 permutations for each test and corrected for multiple comparisons for all voxels in all frequency bands of interest (alpha1, alpha2, gamma). The statistics were shown in decimal logarithm of *F*-ratio values, and the significance threshold was defined to be corrected *p* < 0.050 in the voxel level. All the statistical comparison results were visualized using the BrainNet Viewer toolbox for MATLAB^61^.

### Statistical analysis of discordance in activity and connectivity

Activity level and functional connectivity weights in ROIs were measured for a balance between top-down and bottom-up streams in each hemisphere. The activity level of each stream and hemisphere was added to one representative value. The representative value for the functional connectivity was defined within top-down and bottom-up streams across two of their regions for each hemisphere (i.e. across left frontal eye field and left posterior parietal cortex). The balance of activity or connectivity was evaluated as the interaction between the group effect and the difference between top-down and bottom-up streams. The statistical analysis was performed using the repeated measures ANOVA. The between-subject measure was the group (PD vs. healthy controls), and the within-subject measure was the attention stream. A statistically significant effect was defined as the interaction effect at Bonferroni-corrected *p* < 0.050 (uncorrected *p* < 0.008, corrected for six trials for both activity and connectivity).

### Post-hoc cross-frequency coupling analysis and statistical tests

Based on the results from the previous analyses, a further investigation on cross-frequency coupling of alpha2 and gamma bands was performed. Amplitude-amplitude cross-frequency coupling^45^ was analyzed to verify the functional meaning of narrow-focused attention, which is modulated by alpha2^27^. The time series of the source-localized current signals at alpha2 (10–12 Hz) and gamma (30.5–44 Hz) bands were obtained for top-down ROIs, which consisted of the bilateral posterior parietal cortices and the frontal eye fields. A Hilbert transformation was applied on the band-filtered time series to extract the instantaneous amplitude of the signal. Alpha2 and gamma amplitude values were correlated using bivariate correlation and the resulting coefficients were used to represent the degree of cross-frequency coupling.

For the comparison of cross-frequency coupling across groups, the correlation coefficients between amplitudes created for each top-down ROI were compared between PD and control groups using independent *t*-test. The results were corrected for multiple correction using the Bonferroni correction for four trials. In addition, the coupling values were first correlated to UPDRS 1–3 scores, and the results were corrected for multiple comparisons using the false discovery ratio (FDR) correction for 12 trials^62^. Based on these results, the following test for the correlation between the coupling of bilateral posterior parietal cortices and the subscales within UPDRS 3 was performed in order to determine whether all or some of the subscales contributed to the phenomenon. The correction for multiple comparison was performed using the FDR correction for 28 trials^62^.

### Data Availability

The datasets generated during and/or analyzed during the current study are available from the corresponding author on reasonable request.

## Results

### Participants

The demographic information of the participants is shown in Table 1. PD and control participants were matched for the mean age and the proportion of gender, as previously indicated.

### Whole-brain neural activity analysis

**Figure 2:**
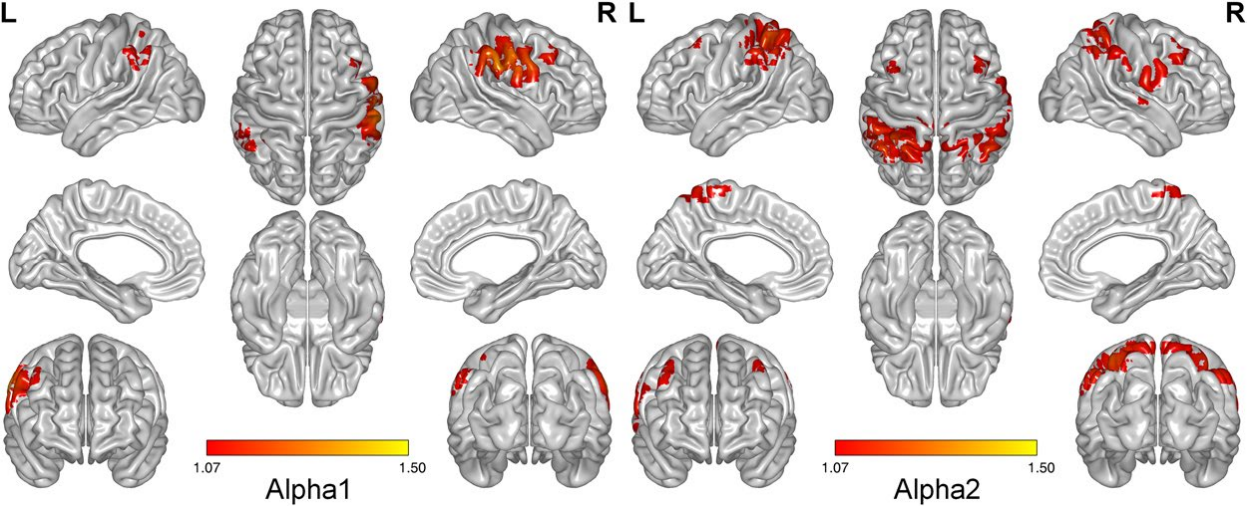
Whole brain subtraction analysis between neural activity levels (current density, unit = log(A/m^2^)) of Parkinson’s disease patients (PD) and healthy control participants (CON). Average activity level in alpha1 and alpha2 bands seems to increase in Parkinson’s disease patients, and the increase is localized in bilateral parietal and lateral frontal regions (two-tailed logarithm of *F*-ratio = 1.07, voxel-level corrected *p* < 0.050). The colorbar represents decimal logarithm of *F*-ratio values ranging from 1.07 to 1.50.

The activity level for all voxels in the brain was compared in PD and control participants to measure the source-localized cortical neural activity. The activity level was significantly higher in the PD group than the control group for alpha1 and alpha2 bands. Figure 2 demonstrates higher neural activity in alpha1 (8–10 Hz) and alpha2 (10–12 Hz) in PD participants (voxel-level corrected *p* < 0.050). The highlighted regions are the bilateral parietal cortices and the lateral frontal regions, which are implied in top-down attention stream. Statistical significance was defined at a two-tailed decimal logarithm of *F*-ratio = 1.07 that was corrected for multiple comparisons (permutation test) at *p* < 0.050. There was no significant change in activity for gamma band (30.5–44 Hz).

### Balance between top-down and bottom-up

**Figure 3:**
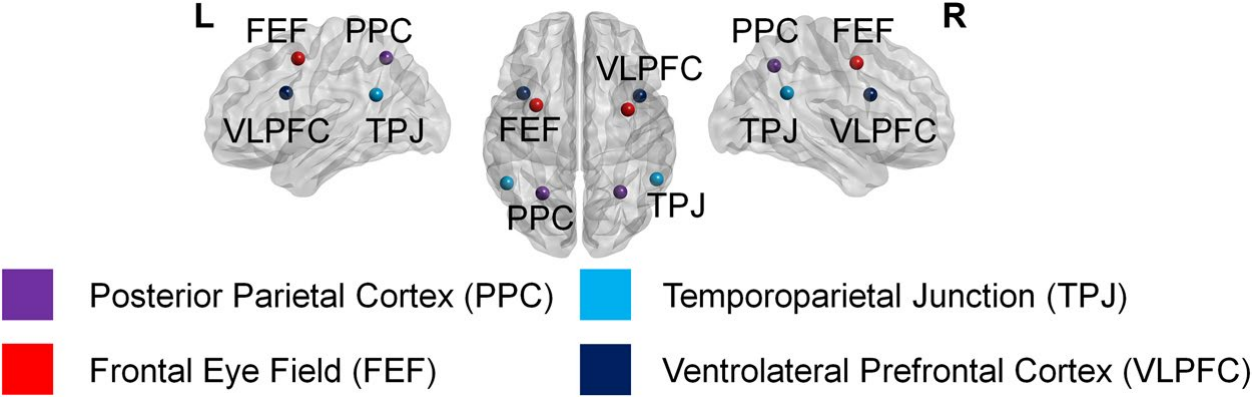
Regions of interest are shown in three-dimensional rendering of brain. Left and right regions implied in top-down (posterior parietal cortices, frontal eye fields) and bottom-up (temporoparietal junctions and ventrolateral prefrontal cortices) attention streams are represented.

**Figure 4:**
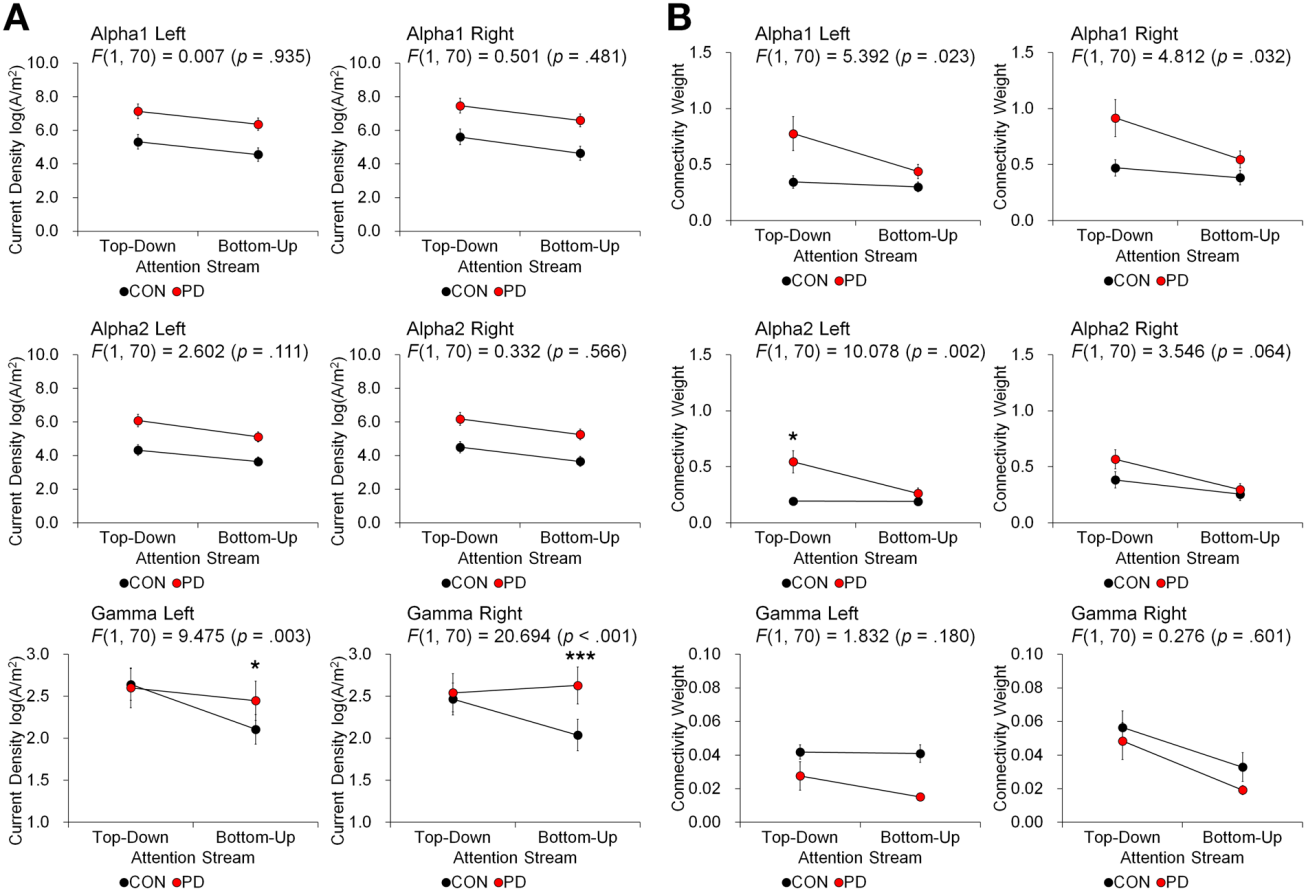
The balance between the neural activity level and functional connectivity in top-down and bottom-up attention streams is represented by the interaction effect from the repeated measures ANOVA. For alpha1, alpha2 and gamma, the balance is shown as the interaction between the group effect and the effect of the attention streams. The reported *p*-values are uncorrected for multiple comparison, but the stars indicate the significance only when the interaction was significant after the Bonferroni correction for six trials (*Bonferroni-corrected *p* < 0.050, ***p < 0.001). Stars are placed on the attention stream showing larger difference between PD and control. The scale is different for gamma bands. Regions of interest are bilateral posterior parietal cortices, frontal eye fields, temporoparietal junctions and ventrolateral prefrontal cortices as defined in Table 2. Error bars represent the standard error values. (**A**) The balance in activity level is shown. In gamma, the interaction effect was significant for both hemispheres, showing that the dominance of activity in top-down stream is stronger in the elderly control compared to Parkinson’s disease patients (PD). (**B**) The balance in the connectivity weights is shown. In alpha2, the interaction effect was significant for the left side, showing that the dominance of connectivity in left top-down stream is stronger in PD.

Regions defined in Table 2 are visualized in Fig. 3. The activity levels summed from regions of top-down and bottom-up streams were compared between the PD and control groups in Fig. 4A. Repeated measures ANOVA showed that there was a significant interaction effect between the group effect and the activity difference of top-down to bottom-up stream for gamma band in both hemispheres, at Bonferroni-corrected *p* < 0.050 (uncorrected *p* < 0.008). The difference in gamma activity across top-down and bottom-up streams in PD was significantly different from the age-matched control. This illustrates the discordance in the activity level in gamma bands for PD patients compared to the control group, which is shown as the disproportionally larger activity in the bottom-up stream in PD, whereas the controls show consistently smaller activity in the bottom-up than top-down stream.

The connectivity weights of top-down and bottom-up streams were compared between the PD and control groups in Fig. 4B. Significant interaction effects between the group effects and the connectivity differences of top-down to bottom-up streams were found in the left hemispheres in alpha2 after the correction for multiple comparisons. The difference in alpha2 connectivity across the left top-down and bottom-up streams in PD was higher than that of the controls, manifesting a disproportionally higher alpha2 connectivity in the top-down stream.

### Cross-frequency coupling comparison

In order to determine whether the cross-frequency coupling of alpha2 and gamma is compensatory or detrimental in top-down stream, the amplitude-amplitude coupling of alpha2 and gamma bands was compared for regions in top-down attention stream between PD and control. Only the group comparison for the right posterior parietal cortex was significant after Bonferroni correction (*t*(55.206) = −2.869, uncorrected *p* = 0.006 < 0.013 for four trials). Comparisons in left posterior parietal cortex (*t*(61.214) = −2.346, *p* = 0.022), left (*t*(61.107) = −1.396, *p* = 0.168) and right frontal eye fields (*t*(69.726) = −1.084, *p* = 0.282) showed no significant differences after multiple correction. The results indicate that the degree of cross-frequency coupling is higher in the right posterior parietal cortex of PD group compared to the control.

### Cross-frequency coupling correlation to Parkinson’s disease symptoms

**Figure 5:**
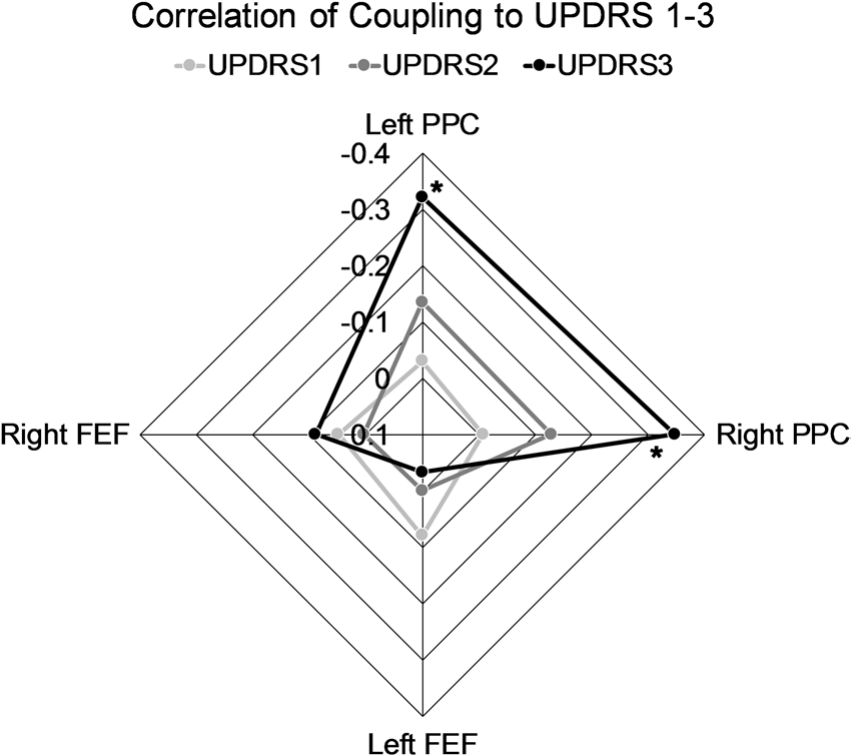
Bivariate correlation of amplitude-amplitude coupling in top-down regions to the Unified Parkinson’s Disease Rating Scale (UPDRS) scores. PPC stands for posterior parietal cortex, and FEF for frontal eye field. The negative correlation of left and right posterior parietal cortices to the motor dysfunction was significant, showing that the higher coupling is, the less severe motor symptoms are in Parkinson’s disease patients (*one-tailed FDR-corrected *p* < 0.050). Please note that the order of correlation values is reversed.

A further analysis was performed by correlating the coupling to the severity of PD symptoms. We tested for a correlation of the coupling of bilateral posterior parietal cortices and frontal eye fields to the UPDRS scores 1–3. Figure 5 shows that there was a significant negative correlation for the coupling to the UPDRS 3 motor scores of the bilateral posterior parietal cortices (FDR-corrected *p* < 0.050). This indicated that the higher coupling of alpha2 and gamma was related to the lower severity of motor symptoms in PD. No significant correlations were obtained with the UPDRS 1 and 2 in these regions.

**Figure 6:**
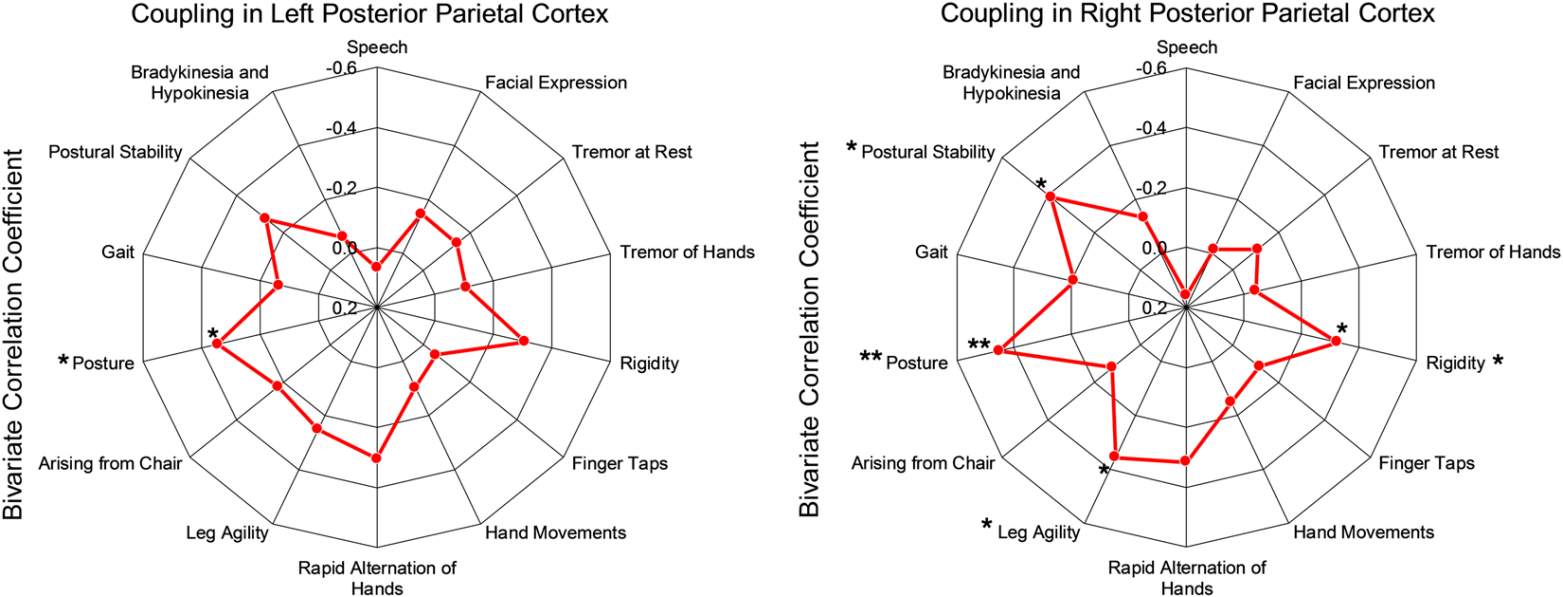
Bivariate correlation of amplitude-amplitude coupling in bilateral posterior parietal cortices to the subscales within Unified Parkinson’s Disease Rating Scale 3 motor scores. The coupling of left posterior parietal cortex was anti-correlated to the severity of posture. The coupling of right posterior parietal cortices was anti-correlated to the severity of symptoms in rigidity, leg agility, posture and postural stability. The negative correlation indicates that the higher coupling is, the less severe motor symptoms are in Parkinson’s disease patients (*one-tailed FDR-corrected *p* < 0.050, **p < 0.010). Please note that the order of correlation values is reversed.

In order to verify if all or some of the UPDRS 3 subscales contributed to the phenomenon, we performed a one-tailed correlation analysis for alpha2-gamma coupling and the UPDRS 3 subscales. Figure 6 shows that there was a significant negative correlation of the coupling in the left posterior parietal cortex to the severity of the posture symptom (uncorrected *p* = 0.020) to the left posterior parietal cortex. It also demonstrated that the coupling in the right posterior parietal cortex was negatively correlated to severity of symptoms in postural stability (*p* = 0.010), posture (*p* = 0.003), leg agility (*p* = 0.017) and rigidity (*p* = 0.026). This indicated that alpha2-gamma coupling in top-down regions may be compensatory for some of the motor symptoms, such as posture and rigidity, but not all of them.

## Discussion

This study is aimed to unravel the neurophysiological signature underlying top-down attention deficits in early and moderate PD. A whole-brain analysis showed that the increase in alpha oscillations was localized to the frontoparietal regions after source localization, revealing that activity changes correspond to the attention network. This study further showed that PD patients have disproportionally higher alpha2 connectivity within the left top-down stream compared to the healthy controls. This tendency was the opposite for gamma activity, for which PD patients showed smaller differences between top-down and bottom-up streams whereas the controls displayed the dominance of gamma activity in top-down over bottom-up. This study further demonstrated that cross-frequency alpha2-gamma coupling was higher in the right posterior parietal cortex for PD, and that the coupling is associated with the lower severity of motor symptoms. The difference in gamma activity and alpha2 connectivity may illustrate the loss of balance between inhibitory modulation (alpha) and excitatory activity (gamma) across top-down and bottom-up attention streams. Alpha and gamma bands seem to exhibit the anti-correlation of power followed by top-down attention demand^37^. To elaborate, alpha bands appear to allocate attention by inhibiting one stream over another^23,24^. It selectively modulates gamma activity directed by top-down attention^36,63^. Altogether, the present results indicate that bottom-up activity is not successfully modulated by inhibitory alpha connectivity on the same stream for PD patients, implying that alpha-gamma modulatory relationship is disproportional in PD, creating the discordance across streams.

We did not find decreases in gamma connectivity for PD when compared to the control group. Dopaminergic medications are found to increase gamma connectivity^64^ by normalizing the connectivity between the premotor and prefrontal regions to restore motor functions^65^. Therefore, it is possible that the lack of difference in gamma between the PD and control was due to the normalization of gamma in PD owing to stable medication^66^. Our study also indicates that medications do not compensate for the discordance of gamma activity between top-down and bottom-up attention streams in PD.

Significant increases of alpha2 connectivity in PD has implications for attentional modulations. The neural activity in alpha band has been related to inhibitory control over sensory information to maintain top-down attention^23,24,67–69^. Alpha plays many roles in maintaining attention in both task-induced and resting states. At rest, alpha sustains an actively alert or a preparatory state for the upcoming demand of selective attention^26,70^. When a task is given, alpha connectivity across top-down stream increases in order to coordinate the attention necessary for the use of working memory^71^. In other words, the dynamics of alpha activity work to alter the state of the brain from a preparatory to a responsive state by releasing the inhibition that was present in the previous state^21,72^. For example, the higher functional connectivity in alpha2 along with the lower gamma activity in the resting state that we found may relate to the inhibitory modulation in the attention streams.

In the present results, PD patients showed disproportionally higher alpha2 connectivity imposing inhibitory modulation over gamma activity in the top-down stream, which may prevent dynamic activation of the stream when on demand. This may be underlying the functional discordance between top-down and bottom-up streams in PD, which manifests as the impairment in top-down attention but intact or even over-activated bottom-up attention^17^. This may imply complex problems in the internal attentional control in PD. It is known that top-down and bottom-up streams continuously interact to optimize attention-related performance^73^ and support complex processing of information^74^. Asplund and coworkers (2010) also showed that the two attention streams are actively interacting and that information relevant to tasks is commonly across the streams. Thus, the over-inhibition of the top-down attention stream compared to less-inhibited bottom-up stream in PD may be related to the inability to balance the over-activated bottom-up system with the relatively under-activated top-down system^17,75^.

The cross-frequency coupling analysis of alpha2 and gamma was performed to verify the implications of these functional discordances. The coupling of alpha2 and gamma was higher in PD for the right posterior parietal cortex, and its value was higher for the lower motor symptoms’ severity (UPDRS 3), but was not significantly associated with other aspects of PD symptoms (UPDRS 1 and 2). It has been suggested that cross-frequency coupling between alpha and gamma is a reflection of attentional processes^76–78^. To note, unlike the functional difference of alpha and gamma manifested in the activity level and connectivity strength, the degree of cross-frequency coupling refers to the communication via gamma modulated by alpha^79,80^. Amplitude-amplitude coupling across frequency bands appears to accommodate the more efficient functional connectivity between distant regions and control neural responses based on attention^81,82^. As alpha-gamma coupling is implicated in the efficient modulation of top-down attention^44,83^, the higher alpha2-gamma coupling in PD may relate to the functional compensation that utilizes increased neural activity in alpha2 band. Alpha2 frequency band (10–12 Hz), in particular, appears to be related to either narrow-focused, goal-directed attention or the alertness that precedes upcoming stimuli^21,26–28^. A recent study showed that mindfulness training improves the motor symptoms of PD patients^84^, and mindfulness training appears to improve the narrow-focused, top-down attention functions^85,86^. This may indicate that the higher alpha2-gamma coupling in PD relates to the functional compensations involving the narrow-focused modulation on top-down attention. Nevertheless, further neurocognitive examinations in future studies are required to confirm the relationship between the alpha2-gamma coupling and the functional changes in PD.

In the additional investigation of whether some or all of the motor symptoms are related to the cross-frequency coupling, higher alpha2-gamma coupling in posterior parietal cortex was found to be correlated to only some of the symptoms, which are posture, rigidity and leg agility within the UPDRS 3, at a lower severity. The posterior parietal cortex appears to encode one’s own body and movement to be used as a postural reference^87,88^, mapping a complex movement in relation to oneself, or following an external model based on motor attention^89,90^. The present results suggest that while higher alpha2-gamma coupling can alleviate some motor symptoms, possibly by aiding narrow attentional control, it does not contribute to symptoms such as bradykinesia and tremors, which are critically influenced by dopaminergic depletion^91,92^.

A limitation of this study is that the participants were not evaluated for behavioral measures related to attention deficits in PD. This study is based on previous literature on selective top-down attention deficits in PD^17,75^, but the association of behavioral changes with neurophysiological evidence will be useful in interpreting our present results in-depth. Studying PD patients in early, non-medicated stages may also help to differentiate the changes in functional connectivity present without external DA supplementation. Furthermore, it is known that the handedness of the subjects can alter the right-hemispheric dominance of the attention streams^93^. Future studies should analyze the confounding effect of handedness upon the attention streams.

In conclusion, this study provides the first electrophysiological evidence that there are discordances in activity and connectivity between top-down and bottom-up attention streams in PD. The disproportionally higher alpha2 connectivity within top-down stream compared to bottom-up may lead to the discordance between two streams, manifested as under-activation of top-down or over-activation of bottom-up in gamma band. However, the interaction between different frequency bands via cross-frequency coupling may relate to functional compensations for PD patients. Higher alpha2-gamma coupling was associated with a lower severity in motor scores of PD patients, suggesting that efficient communication between the two frequency bands might be beneficial for the patients.
